# Risk Factors and Awareness of Preeclampsia Among Pregnant Women Attending Antenatal Care in Nasarawa State

**DOI:** 10.1002/puh2.70285

**Published:** 2026-06-01

**Authors:** Victor Emmanuel, Yohanna Wamanyi, Aisha Ene Umar, Stephen Olaide Aremu, Adamu Ishaku Akyala, Abdillahi Abdi Barkhadle

**Affiliations:** ^1^ Global Health and Infectious Diseases Control Institute Nasarawa State University Keffi, Nasarawa State Nigeria; ^2^ Department of Community Health Nasarawa State University Keffi, Nasarawa State Nigeria; ^3^ Rift Valley University Jimma Ethiopia

**Keywords:** antenatal care, attitude, knowledge, Nigeria, preeclampsia, pregnant women, risk factors

## Abstract

**Background:**

Preeclampsia remains a major contributor to maternal and perinatal morbidity and mortality, particularly in low‐ and middle‐income countries such as Nigeria, where the burden is disproportionately high. Despite increasing awareness, gaps persist in early detection, prevention, and context‐specific evidence, especially in North‐Central Nigeria, including Nasarawa State. This study examined risk factors associated with preeclampsia, antenatal care (ANC) patterns, and pregnant women's knowledge and attitudes toward preeclampsia in selected General Hospitals in Nasarawa State, Nigeria.

**Methodology:**

A descriptive cross‐sectional study was conducted among pregnant women attending antenatal clinics in selected General Hospitals in Nasarawa State. Data were collected using a structured interviewer‐administered questionnaire covering sociodemographic, obstetric, medical, lifestyle, and healthcare access characteristics, as well as knowledge and attitudes toward preeclampsia. Descriptive statistics summarized the variables, while inferential statistics examined associations between selected clinical risk factors and preeclampsia.

**Results:**

A total of 423 pregnant women participated in the study. Most respondents were aged 30–49 years (60.1%) and multiparous (63.4%), with the majority residing in urban areas (88.7%) and having at least secondary education 71.9%. Early ANC initiation before 12 weeks of gestation was reported by 52.2% of participants. A history of chronic hypertension (10.9%) and diabetes mellitus was significantly associated with preeclampsia (*χ* ^2^ = 66.829, *p* < 0.001 and *χ* ^2^ = 423.000, *p* < 0.001, respectively). Knowledge of preeclampsia was high (85.3%), but attitudes were largely neutral (52.0%). Major barriers to ANC utilization included transportation costs (78%), long distance to health facilities (69%), and low health insurance coverage (22.9%). Statistical significance was set at *p* < 0.05.

**Conclusion:**

Clinical risk factors, particularly chronic hypertension and diabetes mellitus, were significantly associated with preeclampsia. Although awareness was relatively high, neutral attitudes toward prevention suggest the need for strengthened health education, early identification of high‐risk pregnancies, and improved access to ANC services.

## Graphical abstract

This study assessed risk factors and awareness of preeclampsia among 423 pregnant women in Nasarawa State, Nigeria. Chronic hypertension and diabetes were significantly associated with preeclampsia. Despite high awareness, preventive attitudes were weak, highlighting the need for improved antenatal education, early risk detection, and better healthcare access.

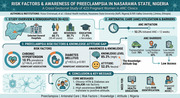

## 1 Introduction

Preeclampsia is a multisystem hypertensive disorder of pregnancy that typically develops after 20 weeks of gestation and is characterized by new‐onset hypertension accompanied by proteinuria or evidence of maternal organ dysfunction [1, 2]. It remains a major cause of maternal and perinatal morbidity and mortality worldwide and is strongly associated with adverse outcomes, including preterm birth, fetal growth restriction, placental abruption, and maternal organ failure [3]. Globally, preeclampsia complicates approximately 2%–8% of pregnancies and is responsible for more than 50,000 maternal deaths and over 500,000 fetal and neonatal deaths each year [4].

The burden is particularly high in low‐ and middle‐income countries (LMICs), especially in sub‐Saharan Africa, where delayed diagnosis, limited access to skilled maternity care, and fragile health systems exacerbate poor maternal and neonatal outcomes [5, 6]. Across Africa, preeclampsia contributes significantly to maternal and pediatric deaths. Reported prevalence includes 9% in Togo, 1% in Rwanda, 10% of maternal fatalities in Ethiopia, and high incidence in Kenya and Zanzibar [7]. Nigeria bears a substantial share of this burden, with hypertensive disorders of pregnancy, including preeclampsia, ranking among the leading causes of maternal mortality in the country [8]. In Nigeria, the condition affects approximately 37,000 women annually and accounts for up to 40% of maternal deaths in Northern Nigeria, with reported prevalence rates ranging from 5.6% to 7.6% in Southern Nigeria and 3.2% in north‐central regions [9, 10, 11].

Despite the growing body of literature, context‐specific evidence on preeclampsia in North‐Central Nigeria, particularly Nasarawa State, remains limited. The high maternal and perinatal mortality associated with preeclampsia in Nigeria has been linked to delays in diagnosis, inadequate access to timely treatment, limited maternal health literacy, and suboptimal antenatal care (ANC) utilization [12, 13]. Regular ANC visits provide opportunities for early detection and monitoring of risk factors such as hypertension and diabetes, as well as for health education on lifestyle modifications and preventive strategies [14, 15]. However, only 20% of pregnant women in Nigeria met the World Health Organization recommendation of at least eight ANC contacts in 2021, highlighting persistent gaps in continuity and quality of maternal healthcare [16].

Antenatal clinics in general hospitals therefore represent critical contact points for the early identification and management of women at risk of preeclampsia. Understanding pregnant women's knowledge, attitudes, and ANC attendance patterns is essential for designing interventions that improve risk stratification, early detection, and preventive care within maternal health services.

This study is conceptually informed by the Health Belief Model (HBM), which explains how individuals’ perceptions of health risks, perceived benefits of preventive actions, and perceived barriers to care influence health‐related behaviors. In maternal health contexts, pregnant women's knowledge of preeclampsia, perceived susceptibility to pregnancy complications, and perceived barriers to accessing ANC may influence their health‐seeking behaviors and preventive practices. Applying this framework helps explain how knowledge, attitudes, and healthcare access factors may affect the early detection and management of preeclampsia among pregnant women [17, 18].

Therefore, this study aimed to examine risk factors associated with preeclampsia, as well as ANC attendance patterns and the timing of the first ANC visit, among pregnant women attending antenatal clinics in selected General Hospitals in Nasarawa State, Nigeria. Specifically, the study examined the influence of sociodemographic, obstetric, medical and clinical risk factors (including hypertension and diabetes mellitus), lifestyle, and healthcare access factors, as well as women's knowledge and attitudes toward preeclampsia, to generate evidence that can strengthen risk stratification and preventive care within antenatal services.

## 2 Methodology

### 2.1 Research Design

The study employed a descriptive cross‐sectional design to examine risk factors associated with preeclampsia among pregnant women attending antenatal clinics at selected General Hospitals in Nasarawa State. This design was deemed appropriate as it enabled the collection of data at a single point in time, providing a comprehensive description of participants’ sociodemographic, obstetric, medical, lifestyle, and healthcare access characteristics, as well as their knowledge of and attitudes toward preeclampsia.

### 2.2 Area of Study

**FIGURE 1:**
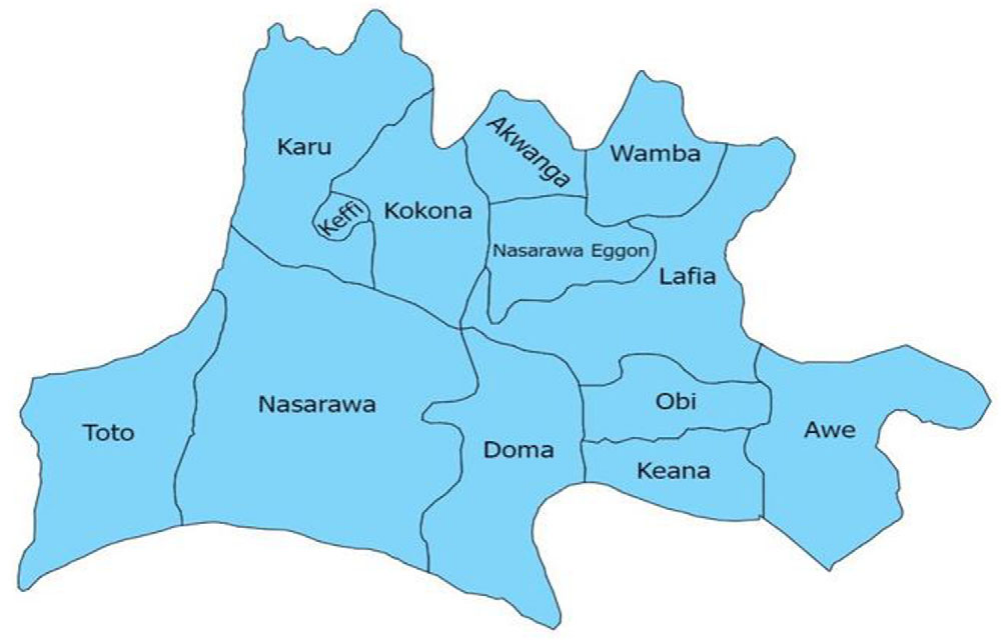
Map of Nasarawa State showing the study area.

The study was conducted in Nasarawa State, North‐Central Nigeria (Figure 1). The state is administratively divided into 13 Local Government Areas and three senatorial zones (North, South, and West) [19]. Public healthcare services are provided through primary, secondary, and tertiary health facilities, including several General Hospitals that provide ANC services for pregnant women. These facilities serve both urban and rural populations and play an important role in maternal healthcare delivery, including screening and management of pregnancy‐related complications such as preeclampsia. Nasarawa State has several public healthcare facilities, including general hospitals offering antenatal services.

### 2.3 Study Population, Sample, and Sampling Techniques

The study population comprised pregnant women attending antenatal clinics at selected General Hospitals in Nasarawa State during the data collection period.

The inclusion criteria for the study population include the following:

1. Pregnant women.
2. Currently receiving ANC at the selected hospitals.
3. Willingness to participate in the study.
4. Residents within the designated study area.

Exclusion criteria:

Pregnant women who were severely ill at the time of data collection, unable to communicate effectively during the interview, or unwilling to provide informed consent were excluded from the study.

The required sample size was calculated using Cochran's formula:

$$\frac{-n=Z^{2}p\left(1-p\right)}{d^{2}}$$

where *Z* = 1.96 (standard normal deviate for 95% confidence level), *p* = 0.5 (estimated proportion for maximum variability), and *d* = 0.05 (margin of error):

$$\frac{n={\left(1.96\right)}^{2}0.5\left(1-0.5\right)=384}{{\left(0.05\right)}^{2}}$$

Accounting for a 10% nonresponse rate: 384+(0.1·384)=423respondents.

Thus, a total of 423 pregnant women were recruited for the study.

### 2.4 Sampling Technique

A multistage sampling approach was employed in this study:

1. Hospital selection: Three general hospitals were purposively selected, one from each senatorial zone (South, North, and West) of Nasarawa State, to ensure geographic representation.
2. Within each hospital, pregnant women attending antenatal clinics were recruited using convenience sampling over a 2‐week period. This method was chosen for practicality and feasibility given the scheduled ANC days.
3. Enrollment: Trained research assistants approached potential participants, explained the study objectives, and obtained informed consent. Eligible women were then provided with a structured questionnaire.

### 2.5 Distribution of Participants Across Facilities

Participants were recruited from three hospitals: General Hospital Keffi, General Hospital Nasarawa Eggon, and General Hospital Doma. The sample was distributed proportionally based on patient volume to ensure representativeness. The distribution of participants across the selected health facilities is presented in Table 1.

**TABLE 1:** Distribution of participants across health facilities.

Sr. no.	Facilities	Sample size
1.	General Hospital Keffi	103 (24.3%)
2.	General Hospital Nasarawa Eggon	165 (39.1%)
3.	General Hospital Doma	155 (36.6%)
	Total	423 (100%)

*Note:* The table presents the distribution of participants across the three hospitals.

### 2.6 Method of Data Collection

Data for this study were collected between September and November 2025 at selected General Hospitals in Nasarawa State using a structured, interviewer‐administered questionnaire developed specifically for this study.

The questionnaire consisted of nine sections:

‐ Section A: Sociodemographic and obstetric characteristics (12 items; multiple‐choice and short‐answer questions).
‐ Section B: Medical history (six items; yes/no questions).
‐ Section C: Lifestyle factors (four items; yes/no and multiple‐choice questions).
‐ Section D: Healthcare access (four items; yes/no, multiple‐choice, and short‐answer questions).
‐ Section E: Clinical data (four items; extracted from participants’ ANC cards/records).
‐ Section F: Determinants of access, awareness, and support (10 items; Likert scale questions).
‐ Section G: Knowledge about preeclampsia (10 items; multiple‐choice questions).
‐ Section H: Attitudes toward preeclampsia and ANC (10 items; 5‐point Likert scale questions).
‐ Section I: Practices related to preeclampsia (10 items; response options: never, rarely, sometimes, often, and always).

#### 2.6.1 Scoring of Knowledge and Attitudes Items

Knowledge items were scored 1 for correct responses and 0 for incorrect responses. Total knowledge scores were computed and categorized as good (≥80%), moderate (50%–79%), or poor (<50%).

Attitude items were measured using a 5‐point Likert scale (strongly agree, agree, neutral, disagree, and strongly disagree). Attitude scores were subsequently categorized as positive, neutral, or negative.

#### 2.6.2 Questionnaire Development and Validation

The questionnaire was developed by the authors on the basis of a review of relevant literature on preeclampsia and maternal health [20, 21].

Content validity was assessed through expert review by three public health specialists with experience in maternal and reproductive health, who evaluated the instrument for clarity, relevance, and appropriateness of the items.

The instrument was pretested among 42 participants (10% of the calculated sample size) at a health facility outside the study sites to assess clarity and comprehensibility. These participants were excluded from the final study sample.

The reliability of the knowledge and attitudes sections was assessed using Cronbach's alpha, which yielded a coefficient of *α* = 0.82, indicating good internal consistency of the instrument.

### 2.7 Outcome Measurement

Preeclampsia status was determined for both previous and current pregnancies. Information on previous history of preeclampsia was obtained through participants’ self‐reports and verified where possible using ANC records. For the current pregnancy, preeclampsia status was verified from participants’ ANC cards/clinical records, where diagnoses made by healthcare professionals were documented based on routine antenatal assessments, including blood pressure measurements and urine protein testing.

### 2.8 Method of Data Analysis

Data were analyzed using IBM SPSS Statistics version 26.0 (IBM Corp., Armonk, NY, USA). Descriptive statistics, including frequencies and percentages, were used to summarize sociodemographic, obstetric, lifestyle, and healthcare access characteristics, ANC attendance patterns, and respondents’ levels of knowledge and attitudes toward preeclampsia. Knowledge items were scored and categorized as good, moderate, or poor, whereas attitudes items were interpreted as positive, negative, or neutral. Responses were recorded on a 5‐point Likert scale (1 = strongly agree to 5 = strongly disagree). Inferential statistical analysis was applied specifically to selected clinical risk factors to examine their association with preeclampsia. Statistical significance was assessed at *p* < 0.05.

### 2.9 Ethical Considerations

Administrative permission was also sought from the management of each participating hospital. Informed consent was obtained from all participants, who were assured of the confidentiality of their responses and their right to withdraw at any time without any repercussions. Appointments for visits to the study sites for questionnaire administration were scheduled in advance to ensure smooth data collection.

## 3 Results

### 3.1 Sociodemographic, Obstetric, and Medical Characteristics of the Respondents

Table 2 presents the sociodemographic, obstetric, and medical characteristics of the 423 pregnant women included in the study. Participants were aged 30–39 years (34.3%), predominantly resided in urban areas (88.7%), were mostly married (93.4%), and had completed secondary education (71.9%). Housewives (40.2%) and business owners (28.1%) were the most common occupations.

**TABLE 2:** Sociodemographic, obstetric, and medical characteristics of pregnant women attending antenatal clinics in selected general hospitals in Nasarawa State (*N* = 423).

Variable	Category	Frequency (*n*)	%	95% CI
**Age group (years)**	20–29	68	16.1	12.6–19.6
	30–39	203	48.0	43.2–52.8
	40–49	152	35.9	31.3–40.5
**Residence**	Urban	375	88.7	85.7–91.7
	Rural	48	11.3	8.3–14.3
**Marital status**	Single	8	1.9	0.6–3.2
	Married	395	93.4	91.0–95.8
	Widowed	5	1.2	0.2–2.2
	Divorced	15	3.5	1.8–5.2
**Education level**	None	28	6.6	4.2–9.0
	Primary	46	10.9	7.9–13.9
	Secondary	304	71.9	67.6–76.2
	Tertiary	45	10.6	7.7–13.5
**Occupation**	Business	119	28.1	23.8–32.4
	SME	82	19.4	15.6–23.2
	Tailor	12	2.8	1.2–4.4
	Housewife	170	40.2	35.5–44.9
	Civil servant	40	9.5	6.7–12.3
**Parity (≥28 weeks)**	1	155	36.6	32.0–41.2
	2	132	31.2	26.8–35.6
	3	85	20.1	16.3–23.9
	4	51	12.1	9.0–15.2
**Gravidity**	1	82	19.4	15.6–23.2
	2	112	26.5	22.3–30.7
	3	103	24.3	20.2–28.4
	4	38	9.0	6.3–11.7
	5	88	20.8	16.9–24.7
**Number of ANC visits**	1	35	8.3	5.7–10.9
	2	115	27.2	22.9–31.5
	3	115	27.2	22.9–31.5
	4	110	26.0	21.8–30.2
	5	48	11.3	8.3–14.3
**History of hypertension**	No	377	89.1	86.1–92.1
	Yes	46	10.9	7.9–13.9
**Family history of preeclampsia**	No	345	81.6	77.9–85.3
	Don't know	58	13.7	10.4–17.0
	Yes	20	4.7	2.7–6.7
**Household monthly income**	<₦30,000	114	27.0	22.8–31.2
	₦30,000–₦50,000	124	29.3	25.0–33.6
	₦51,000–₦100,000	131	31.0	26.6–35.4
	>₦100,000	54	12.8	9.6–16.0
**Gestational age category**	6–13 weeks	61	14.4	11.1–17.7
	14–20 weeks	146	34.5	30.0–39.0
	21–27 weeks	135	31.9	27.5–36.3
	28–33 weeks	81	19.1	15.4–22.8

Abbreviation: ANC, antenatal care.

Regarding obstetric characteristics, most women had one or two previous deliveries, and the majority reported two or three antenatal visits during the current pregnancy. Most participants had no prior history of hypertension (89.1%) and no family history of preeclampsia (81.6%). Household income was largely between ₦30,000 and ₦100,000 per month. Gestational age at data collection ranged from 6 to 33 weeks, with most participants in the second trimester (14–27 weeks).

### 3.2 Lifestyle Characteristics of Respondents

Table 3 presents the lifestyle characteristics of the respondents. Smoking and alcohol use during pregnancy were uncommon, with only 1.9% of participants reporting these behaviors. Regular physical activity was also rare, reported by 1.9% of the women. Fruit and vegetable consumption was more favorable, with 46.1% of respondents reporting daily intake.

**TABLE 3:** Lifestyle characteristics of respondents (*N* = 423).

Variable	Category	Frequency (*n*)	%	95% CI
Smoking during current pregnancy	No	415	98.1	96.8–99.4
	Yes	8	1.9	0.6–3.2
Alcohol use during current pregnancy	No	415	98.1	96.8–99.4
	Yes	8	1.9	0.6–3.2
Physical activity level	No	415	98.1	96.8–99.4
	Yes	8	1.9	0.6–3.2
Frequency of fruit and vegetable intake	Rarely	82	19.4	15.6–23.2
	2–3 times/week	146	34.5	30.0–39.0
	Daily	195	46.1	41.4–50.8

### 3.3 Healthcare Access Characteristics

Table 4 presents healthcare access characteristics of pregnant women attending antenatal clinics in selected General Hospitals in Nasarawa State. Distance to health facilities was a commonly reported barrier, with 38.3% of respondents agreeing and 30.7% strongly agreeing that distance interfered with ANC attendance. Most women reported travel times of 15–30 min (60.8%), whereas 14.2% reported travelling for more than 30 min.

**TABLE 4:** Access and health system factors influencing antenatal care (ANC) attendance (*n *= 423).

Variable	Category	Frequency (*n*)	%	95% CI
Distance from home to clinic is a barrier	Neutral	131	31.0	26.6–35.4
	Agree	162	38.3	33.7–42.9
	Strongly agree	130	30.7	26.3–35.1
Average travel time to hospital	<15 min	106	25.1	21.0–29.2
	15–30 min	257	60.8	56.2–65.4
	>30 min	60	14.2	10.9–17.5
Transportation cost makes ANC difficult	Disagree	12	2.8	1.2–4.4
	Neutral	82	19.4	15.6–23.2
	Agree	214	50.6	45.9–55.3
	Strongly agree	115	27.2	22.9–31.5
Health insurance coverage	No	326	77.1	73.1–81.1
	Yes	97	22.9	18.9–26.9
Clinic staff treat pregnant women respectfully	Neutral	135	31.9	27.5–36.3
	Agree	154	36.4	31.8–41.0
	Strongly agree	134	31.7	27.3–36.1

Transportation costs were a significant financial barrier, with 50.6% of participants agreeing and 27.2% strongly agreeing that transport costs made ANC attendance difficult. Health insurance coverage was low, with only 22.9% of respondents reporting having insurance.

Perceptions of staff attitudes were generally positive, with 68.1% of respondents agreeing or strongly agreeing that clinic staff treated pregnant women respectfully.

### 3.4 ANC Attendance Patterns and Timing of First ANC Visit

Table 5 illustrates the patterns of ANC attendance and timing of the first visit among pregnant women attending selected General Hospitals in Nasarawa State. The results show that more than half of the respondents 52.2% initiated ANC visits before 12 weeks of gestation. A substantial proportion (38.8%) began ANC between 12 and 20 weeks, whereas 9.0% started after 20 weeks, indicating late initiation among a minority of women.

**TABLE 5:** Antenatal care (ANC) attendance patterns and timing of first visit among pregnant women (*n* = 423).

Variable	Category	Frequency (*n*)	%	95% CI
Gestational age at first ANC visit	<12 weeks	221	52.2	47.5–56.9
	12–20 weeks	164	38.8	34.2–43.4
	>20 weeks	38	9.0	6.3–11.7
Number of ANC visits so far	1 visit	35	8.3	5.7–10.9
	2 visits	115	27.2	22.9–31.5
	3 visits	115	27.2	22.9–31.5
	4 visits	110	26.0	21.8–30.2
	5 visits	48	11.3	8.3–14.3

Regarding the number of ANC visits, the data reveal that most women attended between 2 and 4 visits (27.2%, 27.2%, and 26.0%, respectively). Additionally, 11.3% of respondents had already completed five ANC visits, whereas 8.3% attended only one visit at the time of data collection.

### 3.5 Level of Knowledge of Pregnant Women Toward Preeclampsia

**FIGURE 2:**
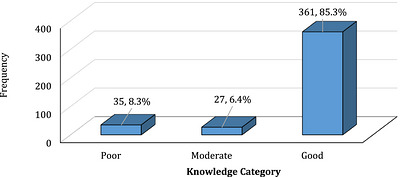
Level of knowledge of pregnant women toward preeclampsia.

Figure 2 shows the distribution of respondents according to their level of knowledge of preeclampsia. The majority of participants demonstrated good knowledge (85.3%), whereas 6.4% had moderate knowledge and 8.3% had poor knowledge.

### 3.6 Attitudes of Pregnant Women Toward Preeclampsia

**FIGURE 3:**
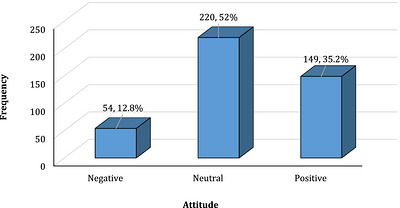
Attitudes of pregnant women toward preeclampsia.

Figure 3 illustrates the distribution of respondents’ attitudes toward preeclampsia. More than half of the participants demonstrated a neutral attitude (52.0%), whereas 35.2% had a positive attitudes and 12.8% exhibited a negative attitudes.

### 3.7 Clinical Risk Factors and Preeclampsia

Table 6 presents the analysis of clinical risk factors and their relationship with preeclampsia among pregnant women attending antenatal clinics. Out of 423 respondents, 46 women (10.9%) reported a history of hypertension. Among these, eight women (17.4%) had experienced preeclampsia in a previous pregnancy, whereas none of the 377 women without hypertension had a history of preeclampsia. The association between hypertension and previous preeclampsia was statistically significant (*χ* ^2^ = 66.829, df = 1, *p* < 0.001).

**TABLE 6:** Association between previous preeclampsia and history of hypertension and diabetes mellitus (*n* = 423).

Previous pregnancy with preeclampsia/eclampsia	Hypertension		Diabetes	
	No (%)	Yes (%)	No (%)	Yes (%)
No	377 (90.8)	38 (9.2)	415 (100.0)	0 (0.0)
Yes	0 (0.0)	8 (100.0)	0 (0.0)	8 (100.0)
*χ* ^2^ (df = 1), *p* value	66.829, <0.001		423.000, <0.001	

Similarly, all 8 women who had preeclampsia in a previous pregnancy also reported a history of diabetes mellitus, whereas none of the 415 women without diabetes experienced preeclampsia. This association was also statistically significant (*χ* ^2^ = 423.000, df = 1, *p* < 0.001).

## 4 Discussion of Findings

### 4.1 Sociodemographic, Obstetric, and Medical Characteristics of the Respondents

The study revealed that most participants were aged 30–49 years (83.9%) and multiparous (63.4%). The majority resided in urban areas (88.7%) and had at least secondary education (71.9%). Most were married (93.4%), and 60.3% reported a monthly income of ≤100,000. A history of hypertension was reported by 10.9% of respondents, whereas only 4.7% had a confirmed family history of preeclampsia.

A substantial proportion of participants were aged 30–49 years. Advanced maternal age (≥35 years) has been associated with an increased risk of preeclampsia [22, 23, 24, 25]. Studies suggest that age‐related changes in cardiovascular function, vascular compliance, and endothelial function may impair maternal adaptation to pregnancy, contributing to the development of hypertensive disorders [26, 27, 28, 29]. Other proposed mechanisms include elevated anti‐angiogenic factors, chronic low‐grade inflammation, and hormonal changes in older women, all of which may compromise placental function and increase preeclampsia risk [30].

Most women in this study were multiparous. Nulliparity has long been recognized as a risk factor for preeclampsia [31, 32]. Both nulliparity and grand‑multiparity were reported as significant predictors of preeclampsia in a recent national review in Nigeria [33]. Immunological mechanisms may partly explain this association, with nulliparous women exhibiting higher levels of circulating anti‐angiogenic factors, such as sFlt1 and an elevated sFlt1/PIGF ratio, which can contribute to the development of preeclampsia [3].

Most respondents resided in urban areas and had at least secondary education (71.9%). Higher education and urban residence are generally associated with better awareness of pregnancy‐related risks and improved access to ANC [34]. Conversely, lower educational attainment has been reported as a significant predictor of preeclampsia in Nigeria, likely due to poorer health literacy, delayed care, and limited engagement with ANC services [33, 35].

A majority of respondents (60.3%) reported a monthly income ≤₦100,000, indicating low socioeconomic status (SES). Low SES has been associated with limited access to healthcare and essential resources, which may contribute to increased risk of preeclampsia [36, 37].

Medical history showed that 10.9% of respondents had chronic hypertension, a well‐established risk factor for preeclampsia due to preexisting vascular pathology and endothelial dysfunction [38]. Chronic hypertension affects approximately 4% of pregnancies and increases the likelihood of adverse outcomes, including preeclampsia, preterm delivery, intrauterine growth restriction, and placental abruption [39]. Studies report that hypertensive women are two to six times more likely to develop preeclampsia compared to normotensive women [40].

Only 4.7% of respondents reported a confirmed family history of preeclampsia, with many unsure. Previous studies indicate that women with a history of preeclampsia have a substantially higher risk of developing the condition in subsequent pregnancies, with reported relative risks ranging from four‐ to five‐fold compared to women without such history [41, 42].

### 4.2 Lifestyle Characteristics of Respondents

In this study, almost all respondents (98.1%) reported abstaining from alcohol and cigarette smoking during pregnancy, indicating a very low prevalence of reported substance use among the study population. This finding is consistent with reports from other Nigerian studies where prenatal substance use has also been reported to be relatively low [43].

Dietary patterns varied among respondents, with 46.1% consuming fruits and vegetables daily, 34.5% consuming them two–three times per week, and 19.4% reporting rare consumption. Previous studies have reported associations between higher fruit and vegetable intake during pregnancy and lower occurrence of hypertensive disorders, including preeclampsia [44, 45, 46].

Physical activity was notably low, with 98.1% of respondents reporting no regular exercise during pregnancy. Evidence from other settings suggests that physical activity during pregnancy is associated with improved cardiovascular and metabolic health [47, 48, 49].

Overall, while substance avoidance and relatively good dietary habits are encouraging, the near‐universal lack of physical activity indicates a critical area for intervention. Promoting culturally appropriate physical activity, alongside dietary counseling, may contribute significantly to reducing the risk of preeclampsia and improving overall maternal health outcomes in this population.

### 4.3 Healthcare Access Characteristics

The findings of this study highlight significant healthcare access barriers that may influence the early detection and management of preeclampsia among pregnant women in Nasarawa State. A substantial proportion of respondents (69%) reported that long travel distances to health facilities hindered their ability to attend ANC. This aligns with findings from other sub‐Saharan African settings, particularly Nigeria, where approximately 75% of women identified distance as a barrier to ANC utilization, contributing to delayed initiation and missed visits, factors known to impede timely diagnosis of hypertensive disorders in pregnancy [50]. Shorter distances to health facilities reduce travel time and transportation costs, thereby facilitating access to necessary care. Ekpenyong et al. [51] further demonstrated that women with travel times of 30 min or less were more likely to make timely healthcare decisions and maintain regular prenatal attendance, particularly when facilities were within walking distance. These findings underscore the importance of improving geographic accessibility of maternal health services to enhance ANC attendance rates.

Transportation‐related constraints were also prominent, with 78% of respondents reporting that transport costs limited their ability to attend ANC. This is consistent with Oyibo et al. [52], who reported transport as a key barrier to timely presentation for hypertensive disorders in Delta State, Nigeria. Similarly, Abdalla et al. [53] identified distance to medical centers and financial constraints as major obstacles to ANC utilization in sub‐Saharan Africa. Across multiple countries, including Nigeria, Mali, Guinea, and Zambia, financial limitations and cultural beliefs remain significant barriers to maternal healthcare access [54]. Wealthier households tend to utilize ANC services more frequently due to affordability, whereas women from lower‐income backgrounds may delay care or miss appointments [55, 56].

Health insurance coverage was markedly low in this study (22.9%), consistent with reports from Jos, Nigeria [57]. Nationally, NHIS coverage remains under 10%, with even lower enrollment among women in the informal sector and rural areas [58, 59]. Health insurance facilitates early ANC initiation and adherence to recommended visit schedules by reducing financial barriers [60]. Conversely, uninsured women are more likely to delay care or avoid health facilities altogether, limiting early detection of high‐risk pregnancies such as preeclampsia [61]. Strengthening sub‐national health insurance schemes, particularly targeting private and informal sector workers, could improve accessibility for vulnerable groups, including pregnant women.

Respectful maternity care is another critical factor influencing ANC utilization. WHO recommends that care should maintain dignity, privacy, and confidentiality, provide freedom from harm, and enable informed choice with continuous support during labor and childbirth [62]. In this study, 68.1% of respondents reported being treated respectfully by clinic staff, similar to rates reported in Calabar (69.9%) [63] and other Nigerian settings [64]. Respectful care encourages continued ANC attendance and timely symptom reporting, whereas the absence of such care may lead women to seek care from unskilled providers, increasing the risk of maternal morbidity and mortality [63].

These findings highlight that physical accessibility, financial capacity, health insurance coverage, and respectful care are key determinants of ANC utilization. Addressing these barriers through targeted policy interventions, improved infrastructure, community‐based health education, and expansion of maternal health insurance schemes could strengthen early detection and management of preeclampsia and other pregnancy complications in Nasarawa State and similar LMIC contexts.

### 4.4 ANC Attendance Patterns and Timing of First ANC Visit

In this study, 52.2% of respondents initiated ANC before 12 weeks of gestation, whereas 38.8% booked between 12 and 20 weeks and 9.0% after 20 weeks. Early ANC initiation, defined as booking during the first trimester in line with WHO recommendations, facilitates timely identification of pregnancy complications and contributes to improved maternal outcomes [62, 65].

Regarding ANC attendance, most women reported two to four visits, with only (11.3%) completing five visits and (8.3%) attending a single visit. This indicates that although initial contact with ANC services was relatively encouraging, continuity of care remained suboptimal. The WHO 2016 ANC model recommends a minimum of eight contacts to reduce perinatal mortality and improve maternal outcomes [66], a target that remains challenging to achieve in many LMICs.

Comparable findings have been reported in Nigeria. A previous study found that 66% of women attended at least four ANC visits [67], whereas the Nigeria Demographic and Health Survey reported that although 67% of women had at least one ANC visit, only 57.4% received optimal care defined as four or more visits [50]. These patterns are consistent with the present study and highlight gaps in continuity of ANC rather than access alone.

### 4.5 Level of Knowledge of Pregnant Women Toward Preeclampsia

In this study, the majority of respondents (85.3%) demonstrated good knowledge of preeclampsia, whereas 6.4% had moderate knowledge, and 8.3% exhibited poor knowledge. This generally high awareness among pregnant women attending antenatal clinics in selected general hospitals in Nasarawa State may reflect prior health education during ANC visits and exposure to maternal health campaigns. In contrast, a study in Ogun State reported that more than 60% of women had never heard of preeclampsia [68], whereas studies in Southwest Nigeria and Kano reported over 60% and 33.9% of women, respectively, with adequate knowledge [69, 70].

The high knowledge levels observed in this study may be partly explained by the relatively high educational attainment of respondents (71.9% with at least secondary education) and the predominance of urban residence (88.7%), which facilitates access to health information. Nonetheless, the presence of poor knowledge in a minority underscores the need for continuous education targeting women with lower educational levels or those residing in rural areas. Effective knowledge dissemination within antenatal clinics is essential to improve early recognition of preeclampsia symptoms and timely ANC attendance, thereby reducing maternal and neonatal complications.

### 4.6 Attitudes of Pregnant Women Toward Preeclampsia

In this study, slightly over half of the respondents (52.0%) held neutral attitudes toward preeclampsia, whereas 35.2% demonstrated positive attitudes, and 12.8% exhibited negative attitudes. Positive attitudes included trust in healthcare advice, willingness to discuss symptoms, and readiness to attend ANC regularly. Neutral attitudes indicate awareness without fully proactive behavior, suggesting that knowledge alone may not always translate into preventive action. Negative attitudes, although less common, may reflect misconceptions, fear, or limited trust in healthcare services.

The proportion of respondents with positive attitudes 35.2% is higher than the 25% reported in a previous Nigerian study [71], but lower than 64% positive attitudes observed in a Tanzanian study [72]. These variations may reflect differences in health education quality, ANC provider communication, and health system responsiveness. Factors influencing attitudes include previous experiences with healthcare providers, sociocultural beliefs, and perceived quality of care [64]. The predominance of neutral attitudes in this study underscores the importance of integrating attitude‐focused interventions into ANC programs, such as counseling, peer support, and culturally appropriate health education, to promote timely recognition and reporting of preeclampsia symptoms.

### 4.7 Clinical Risk Factors and Preeclampsia

The present study demonstrated a statistically significant association between hypertension and a history of preeclampsia among pregnant women attending antenatal clinics. This aligns with existing evidence that hypertensive disorders before or during pregnancy substantially increase the risk of preeclampsia. In Nigeria, Njelita et al. [26] reported that a previous history of pregnancy‐induced hypertension was a strong predictor of preeclampsia (AOR = 76.47; 95% CI: 3.24–1806.29), whereas Aduloju et al. [73] observed that a family history of hypertension significantly increased the likelihood of preeclampsia.

Similarly, this study found a significant association between diabetes mellitus, including gestational diabetes, and preeclampsia. Women with preexisting diabetes are approximately four times more likely to develop preeclampsia compared with non‐diabetic women [74]. Evidence from Nigeria and other African settings supports this finding, highlighting that metabolic disorders contribute to hypertensive complications in pregnancy [63, 75, 76]. The biological relationship is partly explained by shared pathophysiological mechanisms, including endothelial dysfunction, insulin resistance, and angiogenic imbalance [77].

These findings underscore the importance of early risk stratification, intensified monitoring, and targeted preventive interventions for women with underlying hypertensive or metabolic conditions within ANC services.

Early identification and targeted monitoring of high‐risk women, particularly those with chronic hypertension or diabetes, should be prioritized during ANC. Health education programs should not only enhance knowledge but also foster positive attitudes toward preeclampsia, encouraging proactive preventive behaviors. Interventions to reduce barriers to care, including improving geographic access, providing transport support, and expanding health insurance coverage, are critical for optimal ANC utilization [78]. Furthermore, maintaining respectful and supportive maternity care is essential to sustain engagement with healthcare services and ensure timely detection and management of pregnancy complications.

### 4.8 Strengths and Limitations

This study provides a comprehensive assessment of clinical risk factors, ANC patterns, healthcare access, and pregnant women's knowledge and attitudes toward preeclampsia in selected General Hospitals in Nasarawa State. The use of structured interviewer‐administered questionnaires enhanced data completeness and consistency, whereas inferential analysis of key clinical risk factors, particularly hypertension and diabetes mellitus, strengthened the interpretation of associations with preeclampsia.

However, the study has several limitations. The use of Chi‐square tests without multivariable analysis means that potential confounding factors were not controlled for, which may affect the robustness of the observed associations. Additionally, the cross‐sectional design limits the ability to establish causal relationships. The hospital‐based sampling approach may also limit the generalizability of the findings, as it does not capture pregnant women who do not attend ANC services.

Furthermore, some variables, including lifestyle practices, were self‐reported and may be subject to recall or social desirability bias. Finally, inferential analysis was limited to selected clinical risk factors, and findings related to knowledge, attitudes, and healthcare access should therefore be interpreted primarily in descriptive terms.

## 5 Conclusion

This study assessed risk factors and ANC patterns related to preeclampsia among pregnant women attending selected general hospitals in Nasarawa State, Nigeria. Sociodemographic factors, including advanced maternal age, multiparity, urban residence, higher education, and low SES, were associated with both preeclampsia risk and ANC engagement.

Clinical risk factors, notably chronic hypertension and diabetes mellitus, were strongly associated with preeclampsia, underscoring the need for early identification and targeted monitoring. Lifestyle patterns revealed minimal alcohol or tobacco use but very limited physical activity. Healthcare access barriers such as long distances to facilities, transportation costs, and low insurance coverage impeded optimal ANC utilization, whereas respectful maternity care supported engagement.

Although knowledge of preeclampsia was generally high, a substantial proportion of women held neutral or negative attitudes, highlighting that awareness alone may not drive preventive behaviors. These findings underscore the multifactorial determinants of preeclampsia and the importance of integrated antenatal risk assessment, health education, and system‐level interventions to improve maternal outcomes.

## Author Contributions

Victor Emmanuel and Yohanna Wamanyi conceptualized this study. Victor Emmanuel, Yohanna Wamanyi, Aisha Ene Umar, Stephen Olaide Aremu, Adamu Ishaku Akyala, and Abdillahi Abdi Barkhadle conducted a literature search to put together relevant studies. All authors wrote the initial draft, which Victor Emmanuel and Yohanna Wamanyi edited. Abdillahi Abdi Barkhadle is the primary corresponding author, and Victor Emmanuel and Stephen Olaide Aremu are responsible for the work's credibility.

## Funding

The authors have nothing to report.

## Ethics Statement

Ethical approval for the study was obtained from the Health Research Ethics Committee of the Nasarawa State Ministry of Health (Approval No. 05/08/2025). The study adhered to the ethical principles outlined in the Declaration of Helsinki.

## Consent

Consent for participation was collected from all study participants prior to their involvement in the research. Participants were informed of the study's purpose, procedures, and potential risks. They were assured that their participation was voluntary and that their personal information would remain confidential. Written consent was obtained before data collection began.

## Conflicts of Interest

The authors declare no conflicts of interest.

## Data Availability

The datasets generated and analyzed during the current study are not publicly available due to privacy considerations of the participants but are available from the corresponding author upon reasonable request.

## References

[1] E. Dimitriadis , D. L. Rolnik , W. Zhou , et al., “Pre‐Eclampsia,” Nature Reviews Disease Primers 9, no. 1 (2023): 1–22. 10.1038/s41572-023-00417-6 36797292

[2] L. C. Poon , A. Shennan , J. A. Hyett , et al., “The International Federation of Gynecology and Obstetrics (FIGO) Initiative on Pre‐Eclampsia: A Pragmatic Guide for First‐Trimester Screening and Prevention,” International Journal of Gynaecology and Obstetrics 145, no. S1 (2019): 1–33, 10.1002/ijgo.12802. PMC6944283 31111484

[3] K. J. Chang , K. M. Seow , and K. H. Chen , “Preeclampsia: Recent Advances in Predicting, Preventing, and Managing the Maternal and Fetal Life‐Threatening Condition,” International Journal of Environmental Research and Public Health 20, no. 4 (2023): 2994. 36833689 10.3390/ijerph20042994 PMC9962022

[4] T. C. C. Macedo , E. Montagna , C. M. Trevisan , et al., “Prevalence of Preeclampsia and Eclampsia in Adolescent Pregnancy: A Systematic Review and Meta‐Analysis of 291,247 Adolescents Worldwide Since 1969,” European Journal of Obstetrics, Gynecology, and Reproductive Biology 248 (2020): 177–186. 32283429 10.1016/j.ejogrb.2020.03.043

[5] M. Bauserman , V. R. Thorsten , T. L. Nolen , et al., “Maternal Mortality in Six Low‐ and Lower‐Middle‐Income Countries From 2010 to 2018: Risk Factors and Trends,” Reproductive Health 17, no. S3 (2020): 173. 33334343 10.1186/s12978-020-00990-z PMC7745363

[6] World Bank Group, UNDESA/Population Division , Trends in Maternal Mortality 2000 to 2020 (Geneva: World Health Organization, UNICEF, UNFPA, 2023), https://iris.who.int/bitstream/handle/10665/366225/9789240068759_eng.pdf?sequ/ence=1.

[7] P. Vata , N. Chauhan , A. Nallathambi , and F. Hussein , “Assessment of Prevalence of Pre‐Eclampsia From Dilla region of Ethiopia,” BMC Research Notes 8 (2015): 816, 10.1186/s13104-015-1821-5. 26704295 PMC4690301

[8] Z. Mahmoud , I. A. Orji , G. L. Shedul , et al., “Clinical Characteristics and Treatment Patterns of Pregnant Women With Hypertension in Primary Care in the Federal Capital Territory of Nigeria: Cross‐ Sectional Results From the Hypertension Treatment in Nigeria Program,” BMC Pregnancy Childbirth 23, no. 1 (2023): 416, 10.1186/s12884-023-05723-1. 37270521 PMC10239596

[9] T. Olaoye , O. O. Oyerinde , O. J. Elebuji , and O. Ologun , “Knowledge, Perception and Management of Pre‐Eclampsia Among Health Care Providers in a Maternity Hospital,” International Journal of Maternal and Child Health and AIDS 8, no. 2 (2019): 80–88, 10.21106/ijma.279. 31723478 PMC6804318

[10] O. G. Adeosun , M. A. Charles‐Davies , O. A. Ogundahunsi , and J. Ogunlewe , “Maternal and Neonatal Outcome of Pre‐Eclampsia in African Black Women, Southwest Nigeria,” Greener Journal of Medical Sciences 5, no. 1 (2015): 1–8.

[11] P. Awoyesuku , D. John , and L. Lebara , “Maternal and Perinatal Outcome in Severe Preeclampsia and Eclampsia at the Rivers State University Teaching Hospital, Nigeria,” International Journal of Reproduction, Contraception, Obstetrics and Gynecology 9 (2020): 4382–4388, 10.18203/2320-1770.ijrcog20204784.

[12] O. Oladapo , O. Adetoro , B. Ekele , A. Fawole , and I. Morhason‐Bello , “When Getting There Is Not Enough: A Nationwide Cross‐Sectional Study of 998 Maternal Deaths and 1451 Near‐Misses in Public Tertiary Hospitals in a Low‐Income Country,” Bjog 123, no. 8 (2017): 928–938, 10.1111/1471-0528.14447. PMC5016783 25974281

[13] M. M. Obadimeji , T. Olaoye , A. Chikwendu , O. A. Odiari , and R. I. Funwei , “Assessment of the Level of Knowledge and Management Practices of Preeclampsia Among Pregnant Women in Southwest Nigeria,” Pan‐African Journal of Health and Environmental Science 2, no. 2 (2023): 56–69.

[14] C. Nneka , J. Emaimo , and A. J. Emaimo , “Insights Into the Importance of Regular Antenatal Care Visits for Improving Delivery Outcomes in Pregnant Patients: A Cross‐Sectional Study,” European Journal of Science, Innovation and Technology 5, no. 2 (2025): 77–89, https://www.researchgate.net/publication/391978016_Insights_into_the_Importance_of_Regular_Antenatal_Care_Visits_for_Improving_Delivery_Outcomes_in_Pregnant_Patients_A_Cross‐Sectional_Study.

[15] Z. S. Lassi , R. Kumar , and Z. A. Bhutta , “Community‐Based Care to Improve Maternal, Newborn, and Child Health,” in Global Health 2035: A World Converging Within a Generation, ed. R. E. Black , R. Laxminarayan , M. Temmerman , and N. Walker (PubMed Bookshelf, 2019), 299–320. 27227219

[16] A. F. Fagbamigbe , O. Olaseinde , and V. Setlhare , “Sub‐National Analysis and Determinants of Numbers of Antenatal Care Contacts in Nigeria: Assessing Compliance With WHO Recommended Standard Guidelines,” BMC Pregnancy Childbirth 21, no. 1 (2021): 402, 10.1186/s12884-021-03837-y. 34034680 PMC8152343

[17] E. C. Green , E. M. Murphy , and K. Gryboski , “The Health Belief Model,” in The Wiley Encyclopedia of Health Psychology, ed. K. Sweeny , M. L. Robbins , and L. M. Cohen (Wiley, 2020), 211–214, 10.1002/9781119057840.ch68.

[18] A. S. Alamer , “Behavior Change Theories and Models Within Health Belief Model Research: A Five‐Decade Holistic Bibliometric Analysis,” Cureus 16, no. 6 (2024): e63143, 10.7759/cureus.63143. 39055421 PMC11272221

[19] F. A. Akawu and A. Charles , “Impact of Poverty on Access to Healthcare Facilities and Services in Nigeria: A Study of Nasarawa State,” Journal of Economics and Sustainable Development 9, no. 6 (2018): 1–9.

[20] Z. A. Bakori , O. L. Oyetunji , A. E. Abdul , and A. A. Job , “Identifiable Risk Factors and Immediate Outcome of Preeclampsia/Eclampsia in Pregnant Women Managed at Federal Teaching Hospital Katsina, North‐West Nigeria,” Journal of Obstetrics Gynecology and Reproductive Sciences 9, no. 5 (2025): 01–07, 10.31579/2578-8965/272.

[21] F. H. Katore , A. M. Gurara , and T. K. Beyen , “Determinants of Preeclampsia Among Pregnant Women in Chiro Referral Hospital, Oromia Regional State, Ethiopia: Unmatched Case–Control Study,” Integrated Blood Pressure Control 14 (2021): 163–172, 10.2147/IBPC.S336651. 34880674 PMC8646106

[22] N. Sherwan , S. Mubarik , G. Nabi , S. Wang , and C. Fan , “Preeclampsia Mediates the Association Between Advanced Maternal Age and Adverse Pregnancy Outcomes: A Structural Equation Modeling Approach,” Iranian Journal of Public Health 49 (2020): 1727–1733, 10.18502/ijph.v49i9.4092. 33643948 PMC7898110

[23] Z. Naeem , S. Gul , A. Masud , F. Abrar , and T. Z. Dureshahwar , “Association of Pre‐Eclampsia in Women With Advanced Maternal Age,” Journal of Saidu Medical College, Swat 14, no. 1 (2024): 14–18, 10.52206/jsmc.2023.13.4.822.

[24] S. Lisonkova , J. Potts , G. M. Muraca , et al., “Maternal Age and Severe Maternal Morbidity: A Population‐Based Retrospective Cohort Study,” PLoS Medicine 14, no. 5 (2017): e1002307, 10.1371/journal.pmed.1002307. 28558024 PMC5448726

[25] S. D. Smithson , N. H. Greene , and T. F. Esakoff , “Pregnancy Outcomes in Very Advanced Maternal Age Women,” American Journal of Obstetrics & Gynecology MFM 4 (2022): 100491, 10.1016/j.ajogmf.2021.100491. 34543752

[26] I. A. Njelita , C. C. Nwachukwu , G. I. Eyisi , J. C. A. Akabuike , C. A. Ezenyeaku , and C. O. Ifeadike , “Determinants of Preeclampsia in a Tertiary Hospital in South East Nigeria,” International Journal of Medical Science and Clinical Invention 8, no. 6 (2021): 5490–5497, 10.18535/ijmsci/v8i06.08.

[27] R. Dhar , “A Study of Incidence of Pre‐Eclampsia in Relation to Maternal Age,” International Journal of Physiology 9, no. 2 (2021): 23–26, 10.37506/ijop.v9i2.2913.

[28] S. Yagel and S. Verlohren , “Role of Placenta in Development of Pre‐Eclampsia: Revisited,” Ultrasound in Obstetrics & Gynecology: The Official Journal of the International Society of Ultrasound in Obstetrics and Gynecology 56, no. 6 (2020): 803–808, 10.1002/uog.22040. 32275112

[29] C. L. M. Cooke and S. T. Davidge , “Advanced Maternal Age and the Impact on Maternal and Offspring Cardiovascular Health,” American Journal of Physiology. Heart and Circulatory Physiology 317, no. 2 (2019): H387–H394, 10.1152/ajpheart.00045.2019. 31199185

[30] A. C. Eddy , J. A. Howell , H. Chapman , et al., “Biopolymer‐Delivered, Maternally Sequestered NF‐κB Inhibitory Peptide for Treatment of Preeclampsia,” Hypertension 75, no. 1 (2020): 193–201. 31786977 10.1161/HYPERTENSIONAHA.119.13368 PMC7008946

[31] D. Ola and J. Suliburska , “Risk Factors of Preeclampsia in Nigeria and Poland,” Journal of Obstetrics and Gynaecology Research 6, no. 1 (2023): 7–14, 10.5114/jogi.2023.130145.

[32] J. Mayrink , R. T. Souza , F. E. Feitosa , et al., “Incidence and Risk Factors for Preeclampsia in a Cohort of Healthy Nulliparous Pregnant Women: A Nested Case‐Control Study,” Scientific Reports 9 (2019): 9517, 10.1038/s41598-019-45813-3. 31266984 PMC6606578

[33] A. Abdurrahman , A. N. Adamu , A. Ashimi , et al., “Predictors, Prevalence and Outcome of Hypertensive Disorders in Pregnancy in Nigerian Tertiary Health Facilities,” Bjog 131, no. S3 (2024): 42–54, 10.1111/1471-0528.17902. 38960882

[34] U. U. Onyeonoro , O. S. Ogah , A. U. Ukegbu , I. I. Chukwuonye , O. O. Madukwe , and A. O. Moses , “Urban‐Rural Differences in Health‐Care‐Seeking Pattern of Residents of Abia State, Nigeria, and the Implication in the Control of NCDs,” Health Services Insights 9 (2016): 29–36, 10.4137/HSI.S31865.1. 27721654 PMC5053202

[35] E. E. Campbell , J. Gilliland , P. D. N. Dworatzek , B. de Vrijer , D. Penava , and J. A. Seabrook , “Socioeconomic Status and Adverse Birth Outcomes: A Population‐Based Canadian Sample,” Journal of Biosocial Science 50, no. 1 (2018): 102–113. 28270256 10.1017/S0021932017000062

[36] A. C. Efuneshi , S. J. Ozims , and I. F. Eberendu , “Pregnant Women Attending Major Health Facilities in South Eastern Nigeria: A Case‐Control Study on the Socioeconomic Status of Pre‐Eclampsia/Eclampsia,” Global Journal of Research in Medical Sciences 5, no. 1 (2025): 12–20.

[37] J. G. Ray , M. J. Schull , G. Singh , and M. Muhammad , “Socioeconomic Status and Pre‐Eclampsia in a Cohort of Pregnant Women in Canada,” Journal of Obstetrics and Gynaecology Canada 41, no. 6 (2019): 751–758.

[38] C. Martini , Z. Saeed , P. Simeone , et al., “Preeclampsia: Insights Into Pathophysiological Mechanisms and Preventive Strategies,” American Journal of Preventive Cardiology 23 (2025): 101054. 40703703 10.1016/j.ajpc.2025.101054 PMC12284657

[39] A. N. Battarbee , R. G. Sinkey , L. M. Harper , S. Oparil , and A. T. Tita , “Chronic Hypertension in Pregnancy,” American Journal of Obstetrics and Gynecology 222, no. 5 (2020): 532–541. 31715148 10.1016/j.ajog.2019.11.1243

[40] C. T. Nguefack , M. A. Ako , A. T. Dzudie , T. N. Nana , P. N. Tolefack , and E. Mboudou , “Comparison of Materno‐Fetal Predictors and Short‐Term Outcomes Between Early and Late Onset Pre‐Eclampsia in the Low‐Income Setting of Douala, Cameroon,” International Journal of Gynaecology and Obstetrics 142, no. 2 (2018): 228–234, 10.1002/ijgo.12531. 29761476

[41] J. Musa , C. Mohammed , A. Ocheke , M. Kahansim , V. Pam , and P. Daru , “Incidence and Risk Factors for Pre‐Eclampsia in Jos,” Nigeria African Health Sciences 18, no. 3 (2018): 584–595, 10.4314/ahs.v18i3. 30602991 PMC6307024

[42] S. Singh , E. Ahmed , S. Egondu , and N. Ikechukwu , “Hypertensive Disorders in Pregnancy Among Pregnant Women in a Nigerian Teaching Hospital,” Nigerian Medical Journal 55, no. 5 (2018): 384–388, 10.4103/0300-1652.140377. PMC4178334 25298602

[43] I. A. Adeoye , “Alcohol Consumption and Tobacco Exposure Among Pregnant Women in Ibadan, Nigeria,” BMC Psychiatry 22 (2022): 570, 10.1186/s12888-022-04210-9. 36002900 PMC9400274

[44] T. Grum , S. Hintsa , and G. Hagos , “Dietary Factors Associated With Preeclampsia or Eclampsia Among Women in Delivery Care Services in Addis Ababa, Ethiopia: A Case‐Control Study,” BMC Research Notes 11, no. 1 (2018): 3793–3798, 10.1186/s13104-018-. PMC6167851 30285827

[45] M. Endeshaw , F. Abebe , M. Bedimo , and A. Asart , “Diet and Pre‐Eclampsia: A Prospective Multicentre Case‐Control Study in Ethiopia,” Midwifery 31, no. 6 (2015): 617–624, 10.1016/j.midw.2015.03.003. 25862389

[46] B. Mi , X. Wen , and S. Li , “Vegetable Dietary Pattern Associated With Low Risk of Preeclampsia Possibly Through Reducing Proteinuria,” Pregnancy Hypertens 16 (2019): 131–138, 10.1016/j.preghy.2019.04.001. 31056148

[47] N. Ferrari and C. Joisten , “Impact of Physical Activity on Course and Outcome of Pregnancy From Pre‐ to Postnatal,” European Journal of Clinical Nutrition 75 (2021): 1698–1709, 10.1038/s41430-021-00904-7. 33828239 PMC8636258

[48] H. A. Pahlavani , I. Laher , K. Weiss , B. Knechtle , and H. Zouhal , “Physical Exercise for a Healthy Pregnancy: The Role of Placentokines and Exerkines,” Journal of Physiological Sciences 73, no. 1 (2023): 30, 10.1186/s12576-023-00885-1. PMC10718036 37964253

[49] American College of Obstetricians and Gynecologists , “Physical Activity and Exercise During Pregnancy and the Postpartum Period: ACOG Committee Opinion No. 804,” Obstetrics and Gynecology 135, no. 6 (2020): e178–e188. 32217980 10.1097/AOG.0000000000003772

[50] O. K. Oyedele , “Disparities and Barriers of Health Facility Delivery Following Optimal and Suboptimal Pregnancy Care in Nigeria: Evidence of Home Births From Cross‐Sectional Surveys,” BMC Womens Health 23, no. 1 (2023): 194, 10.1186/s12905-023-02364-6. 37098533 PMC10131351

[51] M. S. Ekpenyong , D. Matheson , and L. Serrant , “The Role of Distance and Transportation in Decision Making to Seek Emergency Obstetric Care Among Women of Reproductive Age in South‐South Nigeria: A Mixed Methods Study,” International Journal of Gynaecology and Obstetrics 159, no. 1 (2022): 263–269, 10.1002/ijgo.14103. 35044678 PMC9545747

[52] P. Oyibo , O. Uwomano , K. O. Obohwemu , I. F. Ndioho , E. O. Eke , and E. M. Umuerri , “Barriers and Enablers of Antihypertensive Adherence Among a Nigerian Adult Hypertensive Population Seeking Care in Public Secondary Health Facilities in Delta State, Nigeria: A Mixed Methods Study,” West African Journal of Medicine 42, no. 3 (2025): 240–247. 40845429

[53] S. Abdalla Osman Mohamed , G. S. Mohamed , A. K. Attaelmanan Mahgoub , et al., “Barriers to Antenatal Care Attendance in Developing Countries: A Systematic Review,” Cureus 17 (2025): e95342, 10.7759/cureus.95342. 41287658 PMC12640685

[54] B. O. Ahinkorah , E. K. Ameyaw , A. A. Seidu , E. K. Odusina , M. Keetile , and S. Yaya , “Examining Barriers to Healthcare Access and Utilization of Antenatal Care Services: Evidence From Demographic Health Surveys in Sub‐Saharan Africa,” BMC Health Services Research [Electronic Resource] 21 (2021): 125, 10.1186/s12913-021-06129-5. 33549089 PMC7866461

[55] Y. Abdullahi and Z. Umar , “Socio‐Economic Barriers to Antenatal Care Utilization in Northern Nigeria,” Journal of Public Health in Africa 14, no. 3 (2020): 55–66.

[56] A. Adeyemi , “Cultural Determinants of Antenatal Care Attendance in Northern Nigeria,” African Journal of Health Sciences 15, no. 2 (2021): 103–114.

[57] F. A. Magaji , J. M. Ali , W. Golit , et al., “National Health Insurance Scheme Coverage for Pregnant Women in Jos, Nigeria: Implications for SDG‐3,” Journal of Health Sciences and Practice 1, no. 1 (2022): 26–31.

[58] W. Adekunle and O. Vincent , “Analysing the Determinants of Healthcare Insurance Uptake in Nigeria,” BMC Health Services Research [Electronic Resource] 25, no. 1 (2025): 1310, 10.1186/s12913-025-13422-0. 41053776 PMC12502249

[59] A. I. Okpani and S. Abimbola , “Lessons From the Implementation of NHIS in Nigeria: A Critical Policy Analysis,” BMC Health Services Research [Electronic Resource] 23, no. 1 (2023): 57.36658517

[60] O. P. Ezeoke , R. Okonkwo , and N. Nwachukwu , “Impact of Health Insurance on Antenatal Care Attendance and Maternal Health Outcomes in Nigeria,” African Health Sciences 22, no. 4 (2022): 878–886.

[61] E. Nwankego‐Obeya , “Assessing the Effectiveness of the National Health Insurance Scheme (NHIS) on Maternal Health Outcomes in Jos Metropolis, Nigeria,” International Journal of Humanities Social Science and Management 5, no. 3 (2025): 200–214.

[62] World Health Organization , WHO Recommendations on Antenatal Care (WHO, 2018).

[63] M. E. John , E. U. Duke , and E. E. Esienumoh , “Respectful Maternity Care and Midwives' Caring Behaviours During Childbirth in Two Hospitals in Calabar,” Nigeria African Journal of Biomedical Research 23, no. 1 (2020): 165–169.

[64] A. N. Adamu and A. G. Umar , “Respectful Maternity Care Practice: An Assessment of Parturients' Experience at Federal Medical Center, Birnin Kebbi, Nigeria,” Tropical Journal of Obstetrics and Gynaecology 42, no. 1 (2024): 24–28.

[65] World Health Organization , Maternal Mortality: Key Facts (WHO, 2019), https://www.who.int/newsroom/fact‐sheets/detail/maternal‐mortality.

[66] World Health Organization , WHO Recommendations on Antenatal Care (WHO, 2016).

[67] M. F. Olanrewaju , “Analysis of Knowledge and Practice of the New Antenatal Care Guidelines Among Pregnant Women Attending Antenatal Clinics in Surulere Area,” IOSR Journal of Dental and Medical Sciences 18, no. 10 (2019): 23–29.

[68] O. M. Oluseye , F. R. Olowolagba , O. P. Olatunji‐Adewunmi , and F. M. Akinsoji , “Knowledge and Perception of Pre‐Eclampsia and Eclampsia Among Pregnant Women Attending Antenatal Clinic in a State Hospital, Ogun State, Nigeria,” Nigerian Health Journal 23, no. 4 (2023): 952–960, 10.60787/tnhj-752.

[69] S. A. Oladosu , P. O. Okimi , and E. O. Oladosu , “Perceived Impacts of Pre‐Eclampsia and Eclampsia on Health of Pregnant Mothers Being Attended to at a General Hospital in Southwest,” International Clinical Pathology Journal 9, no. 1 (2022): 42–45.

[70] U. Igbokwe , U. Tukki Tsikasom , U. Korfii , et al., “Knowledge, Attitude and Practice of Pre‐Eclampsia/Eclampsia Preventive Measures Among Pregnant Women in Selected Primary Health Care Facilities in Kano State: A Cross‐Sectional Study,” Research Square 14 (2024): 4431–4445, 10.21203/rs.3.rs-4034471/v1. PMC12704996 41403506

[71] T. Chanda , “Knowledge, Attitudes, and Beliefs About Pre‐Eclampsia and Eclampsia Among Pregnant Women at Selected Antenatal Clinics in Kitwe,” Journal of Clinical Research 7 (2023): 194.

[72] D. E. Ejike , B. Ambrose , and D. A. Moses , “Determination, Knowledge and Prevalence of Pregnancy‐Induced Hypertension/Eclampsia Among Women of Childbearing Age at Same District Hospital in Tanzania,” International Journal of Medical Sciences 10, no. 2 (2018): 19–26.

[73] O. P. Aduloju , A. Fajola , and S. D. Tuboteme , “Prevalence, Risk Factors and Maternal‐Fetal Outcomes of Hypertensive Disorders in Pregnancy: A 5‐Year Retrospective Review at a Cottage Hospital in Rivers State, Nigeria,” Sierra Leone Journal of Medicine 2, no. 1 (2025): 1–6.

[74] N. C. Do , M. Vestgaard , S. K. Nørgaard , P. Damm , E. R. Mathiesen , and L. Ringholm , “Prediction and Prevention of Preeclampsia in Women With Preexisting Diabetes: The Role of Home Blood Pressure, Physical Activity, and Aspirin,” Frontiers in Endocrinology 14 (2023): 1166884, 10.3389/fendo.2023.1166884. 37614711 PMC10443220

[75] A. S. Belay and T. Wudad , “Prevalence and Associated Factors of Pre‐Eclampsia Among Pregnant Women Attending Antenatal Care at Mettu Karl Referral Hospital, Ethiopia: A Cross‐Sectional Study,” Clinical Hypertension 25, no. 1 (2019): 14, 10.1186/s40885-019-0120-1. 31304042 PMC6600877

[76] O. I. Hounkpatin , S. A. Amidou , Y. C. Houehanou , et al., “Systematic Review of Observational Studies of the Impact of Cardiovascular Risk Factors on Preeclampsia in Sub‐Saharan Africa,” BMC Pregnancy Childbirth 21 (2021): 97, 10.1186/s12884-021-03566-2. 33516185 PMC7847133

[77] K. Pankiewicz , E. Szczerba , A. Fijałkowska , J. Sierdziński , T. Issat , and T. M. Maciejewski , “The Impact of Coexisting Gestational Diabetes Mellitus on the Course of Preeclampsia,” Journal of Clinical Medicine 11, no. 21 (2022): 6390, 10.3390/jcm11216390. 36362618 PMC9655937

[78] Y. M. Suleiman , W. Yohanna , S. O. Aremu , G. I. Halidu , and A. I. Adamu , “Utilization, Satisfaction, and Perceived Maternal Health Benefits of Group Antenatal Care in Karu LGA, North Central, Nigeria,” BMC Pregnancy Childbirth 26, no. 1 (2025): 73, 10.1186/s12884-025-08579-9. 41408528 PMC12829021

